# Fostering shared decision-making about prostate cancer screening among African American men patients and their primary care providers: a randomized behavioral clinical trial

**DOI:** 10.1186/s13063-022-06605-1

**Published:** 2022-08-13

**Authors:** Margarita Echeverri, Kyazia Felder, David Anderson, Elora Apantaku, Patrick Leung, Clare Hoff, Princess Dennar

**Affiliations:** 1 Educational Coordinator Health Disparities, Diversity and Cultural Competence; Center of Minority Health and Health Disparities Research and Education, Xavier University of Louisiana; College of Pharmacy, 1 Drexel Drive, New Orleans, LA 70125 USA; 2 Department of Clinical and Administrative Sciences, Xavier University of Louisiana, 1 Drexel Drive, New Orleans, LA 70125 USA; 3 Department of Mathematics, Xavier University of Louisiana, 1 Drexel Drive, New Orleans, LA 70125 USA; 4 Preventive Medicine Residency PGY2, Tulane University, School of Medicine, 131 S. Robertson St, 11th Floor, New Orleans, LA 70112 USA; 5 Medical Physicians Group PLLC, 11555 Central Parkway, Suite 903, Jacksonville, FL 32224 USA; 6 Department of Family and Community Medicine, Tulane University School of Medicine, 131 S. Robertson St, 11th Floor, New Orleans, LA 70112 USA; 7 Premium Care Medical Center, LLC, 3570 Holiday Drive, Suites 3-7, New Orleans, LA 70114 USA

**Keywords:** Prostate cancer, PSA screening, African American men, Shared decision-making, Decision aid, Primary care providers, Randomized controlled trial

## Abstract

**Background:**

Prostate cancer is the third most prevalent cancer in the American population. Furthermore, the prognosis is worse in African American as there is increased morbidity and mortality associated with it.

**Purpose:**

The purpose of this study is to evaluate the effectiveness of a new online method to educate the patient population regarding prostate cancer risk, diagnosis, treatments, and their decisions about whether to be screened for the early detection of prostate cancer.

**Methods:**

Two hundred Black male patients are recruited from different clinical sites and randomized to either the control arm (usual care) or the intervention arm (educational program). We will compare the effectiveness of the intervention to see if patients are discussing the need of getting a prostate-specific antigen (PSA) test, and the possible benefits and harms that may result of having or not having the test, with their primary care providers.

**Discussion:**

Shared decision-making (SDM) is the current standard in most cancer-screening guidelines and also a standard of person-centered care. However, there is a lack of evidence-based approaches to improve decision quality in clinical settings and an increased ambiguity of applying SDM for PSA-based screening among Black men in primary care. Our proposal to evaluate a decisional-aid intervention and measure the actual application of SDM during clinical encounters has a high potential to advance the translation path of implementing shared decision-making in clinical settings and provide evidence of the applicability of the guideline in general.

**Innovation and overall impact:**

Given the 2018 USPSTF updated guidelines recommending shared decision-making about PSA-based screening, the increased risk of prostate cancer mortality in Black men, the challenges of evidence-based decision-making due to the underrepresentation of Blacks in major randomized clinical trials, and implicit racial bias among primary care providers, the time is ripe for interventions to improve shared decision-making about prostate cancer screening in Black men. In this study, we address communication and knowledge gaps between Black men and their primary care providers. The intervention, if proven effective, can be readily scaled across primary care practices across the U.S. and may be adapted to other types of cancer where guidelines have included shared decision-making as well. Early detection of prostate cancer may decrease mortality and morbidity in the long term.

**Supplementary Information:**

The online version contains supplementary material available at 10.1186/s13063-022-06605-1.

## Background

Despite years of aggressive prostate cancer (PrCa) screening practices and declines in mortality in the U.S., African American men (AAM) still have the highest PrCa incidence and mortality rates. Not only is PrCa the most common cancer in AAM but a substantial proportion of AAM have an earlier age of onset, increased proportion of clinically advanced disease, and increased mortality from PrCa compared to White American men [1]. Louisiana has the 2nd highest incidence and 7th highest mortality rate of PrCa in the U.S. [2, 3]. Although African Americans in the U.S. represent 13.4.6% of the total population, they comprise 33% of the Louisiana population and 60% of the population in Orleans Parish [4].

The 2018 U.S. Preventive Services Task Force (USPSTF) prostate cancer screening guidelines recommend that providers and patients should engage in shared decision-making about prostate-specific antigen (PSA)-based screening [5]. Through shared decision-making, primary care providers (PCPs) can empower patients to understand their personal risk and the benefits, harms, and uncertainty of PSA-based screening to make an individualized decision as to whether screening is right for them. As indicated in the 2018 guidelines, shared decision-making is particularly important for AAM patients given the elevated risk of prostate cancer incidence and mortality [1]. SDM is also included as a standard of person-centered care in clinical practice [6], and it is in the list of recommended improvement activities in the Merit-based Incentive Payment System (MIPS), Quality Payment Program [7]. Shared decision-making is anticipated to improve the overall decision-making process, meaning that patients will gain increased knowledge about screening, increased decisional confidence, and increased decision satisfaction. Moreover, as emphasized by the USPSTF, the SDM process can help patients make preference-congruent decisions, meaning that the decision to have or avoid PSA-based screening will be driven by patient preferences rather than providers’ default practices or implicit biases in primary care. However, there is a lack of PSA-based research regarding the benefits and harms of PSA-based screening specific to this population. In general, AAM were underrepresented in the two major randomized clinical trials (RCTs) evaluating PSA-based screening [5], AAM have more barriers to PSA screening and misconceptions about PrCa aggressiveness and cure [8], and SDM conversations about PSA screening are less common for African American than White American male patients [9].

Consequently, in order to implement the guidelines, there is a need to evaluate the efficacy of an intervention to improve shared decision-making about PSA-based screening, especially among AAM patients in the New Orleans area. The protocol follows the Standard Protocol Items: Recommendations for Interventional Trials (SPIRIT) guidelines and fulfills the SPIRIT checklist (see Additional file 1: SPIRIT Checklist).

## Objectives

The proposed study is a behavioral randomized clinical trial (RCT) to advance the translation path of implementing the shared decision-making (SDM) process regarding PSA-screening during clinical encounters. This research aims to examine the impact of the intervention on African American men (AAM) patients’ use of shared decision-making (SDM) with their primary care providers when discussing whether they want or not to have the prostate-specific antigen (PSA)-based screening test for early detection of prostate cancer (primary outcome).

The RCT involves evaluating the efficacy multimedia intervention for AAM conducted before a clinical encounter to increase shared decision-making about PSA screening versus usual care. As the trial’s intervention focuses only on delivering an educational component to participants in the intervention arm, there is not alteration to usual care pathways (including use of any medication or clinical exams) for either of the trial arms. Table 1 includes the list of study aims, outcomes, measures, research questions, and hypotheses.

**Table 1:** Study aims and expected outcomes

Aim	Outcome	Measure^**a**^	Research question	Hypothesis
**Aim 1: Shared decision-making (SDM)**: Assess the efficacy of the intervention in increasing provider and patient engagement in SDM	**Primary clinical outcome:** Level of engagement in SDM process regarding PSA-based screening	Rating of audio-recorded medical visits using the Observing Patient Involvement (OPTION) Scale [10] and patients and providers self-assessment of SDM [11, 12]	Does the intervention increase patient-provider engagement in SDM?	Patients in the intervention arm will demonstrate more engagement (higher scores) in the SDM process than patients in the control arm
**Aim 2: Quality of Decision:** Assess the efficacy of the intervention in improving the quality of the provider-patient SDM process	**Secondary outcomes:** Patient Quality of Engagement in Decision-Making (PQED) regarding PSA-based screening	Change in pre-posttests using an average difference in pre-posttest scores of four subscales: • Prostate Cancer and Screening Knowledge [13] • Decisional Confidence [14] • Decisional Self-efficacy [15] • Satisfaction with Decision [16]	Does the intervention increase patients’ knowledge, decision confidence, satisfaction, and self-efficacy regarding PSA-screening decisions?	Patients in the intervention arm will report higher change between pre-posttests scores, than patients in the control arm
	Preference-Congruent Decision-making (PCDM)	Comparison score between the intention-to-screen [17] and data on actual PSA-tests extracted from the patient electronic health record	Does the intervention increase preference-congruent decision-making regarding PSA-screening?	Patients in the intervention arm will report higher congruence between intention-to-screen and real action, than patients in the control arm
**Evaluation of study:** Evaluate the acceptability of intervention and study procedures	**Exploratory outcomes:** Acceptability of intervention (decision aid) and study procedures (recruitment, assessments, and medical encounter)	Summed score of the adapted version of three measures [18]: **•** Intervention Measure (AIM) **•** Intervention Appropriateness Measure (IAM) **•** Feasibility of Intervention Measure (FIM)	Were study procedures and intervention accepted by participants?	A high percentage of participants will rate the study procedures and intervention as acceptable, or highly acceptable

^a^Measures used in this study have been adapted from the ones found in the literature

## Study design

This is a behavioral randomized clinical trial, superiority parallel two-arm design with 1:1 allocation ratio. The study will compare results from an educational intervention versus usual care. A total of 200 AAM patients are randomly assigned to receive the intervention (*n* = 100) or usual care (*n* = 100). The intervention consists of multimedia educational training materials to increase patients’ understanding of prostate cancer (risk, screening, diagnoses, and treatment), PSA-based screening (benefits, risks, harms, and guidelines), and shared decision-making process (options, perceptions, preferences, actions). All patients complete a series of surveys in several intervals during the study period to collect pre-post and follow-up data to assess study outcomes: at baseline (visit 1), immediately after the patient-PCP encounter (visit 2), and at 3 months after the patient-PCP encounter (+ 15 days; visit 3). Additionally, PCPs and patients discuss, during the clinical encounter, PSA-based screening, and the conversations are audio-recorded and rated to assess the application of SDM during the encounter. Generalized linear models will be used to test our hypotheses that (1) the intervention will increase provider-patient engagement in SDM, and (2) patients in the intervention arm will have higher knowledge, decision confidence, self-efficacy, satisfaction, and preference-congruent decision making than those in the control arm. ANOVAs and chi-square tests will assess group differences on PCPs’ and patients’ characteristics.

**Fig. 1:**
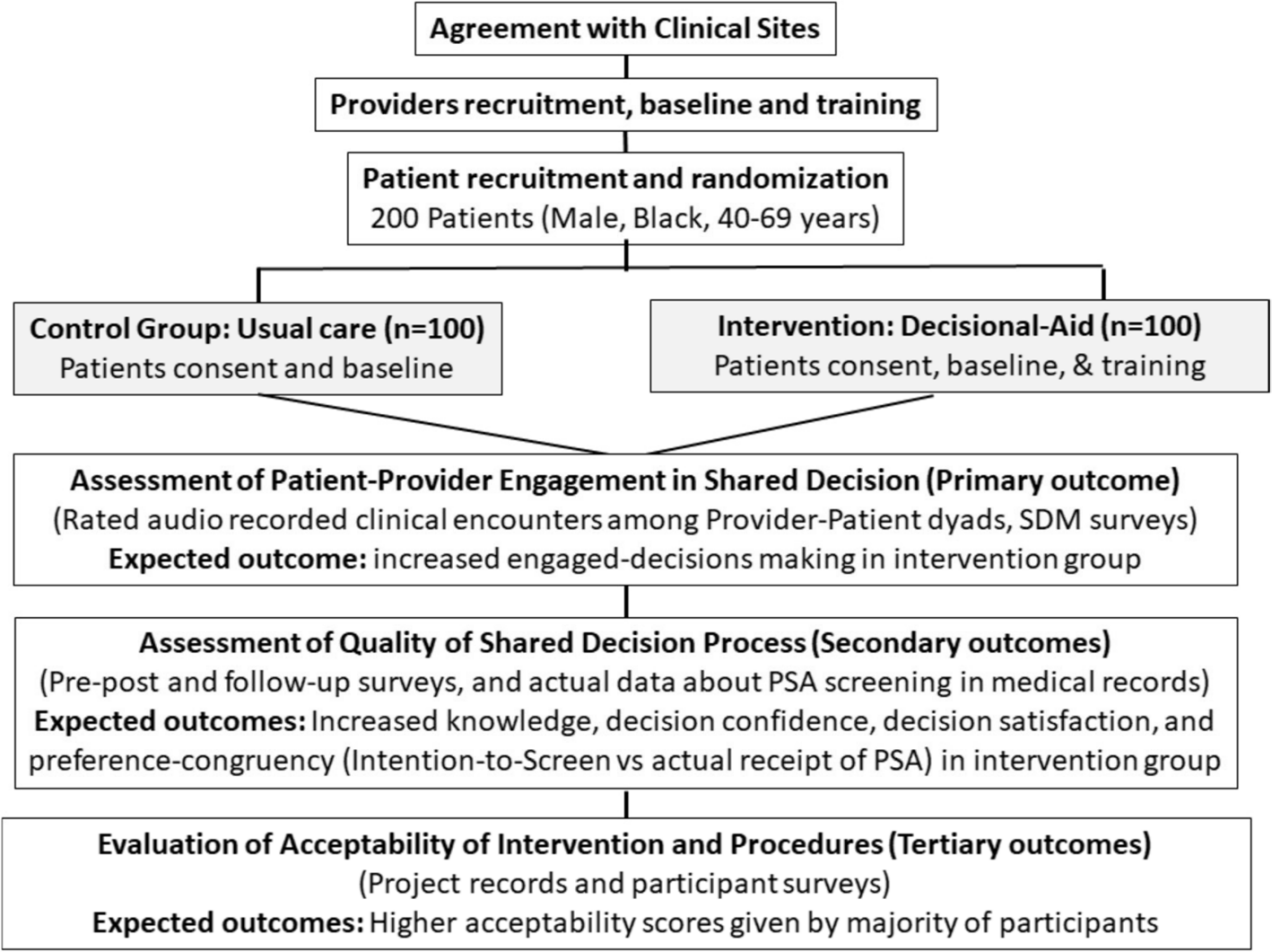
Study design schema

The study design schema about the randomization of patients and assessment for both the intervention and the control arms is shown in Fig. 1.

### Sample size calculation

The RCT involves 200 African American male (AAM) patients who are receiving primary care at the study clinical sites during the study timeline. The proposed study is powered to evaluate the hypotheses of all aims and associated sensitivity analysis [19]. All power calculations and associated sensitivity analyses were computed using the statistical power analysis program G*Power, version 3.1.3 [20], assume a two-tailed alpha level of 0.05, and include covariates as specified in the “Statistical analysis” section.

The main explanatory variables compare patients in the intervention arm (those who received an intervention designed to improve the decision-making process) to patients in the control arm (usual care). The main response variable (primary outcome) is the OPTION score, which measures the level of engagement in shared decision-making using ratings for the analysis of audio recordings (Table 1). The OPTION score is a numerical measure ranging from 0 to 48, with higher scores indicating better engagement in shared decision-making. Since we are comparing two independent arms (intervention and control patients) on a numerical primary outcome (OPTION score), the main interest is in the power of a two-tailed *t*-test comparing means for two independent groups. We aim for power between 80% and 95% (*β* = 0.05 to *β* = 0.2), and an effect size of *d* = 0.5, able to detect differences of half a standard deviation between the means, which is considered a moderate effect size, or “one large enough to be visible to the naked eye” [21].

**Fig. 2:**
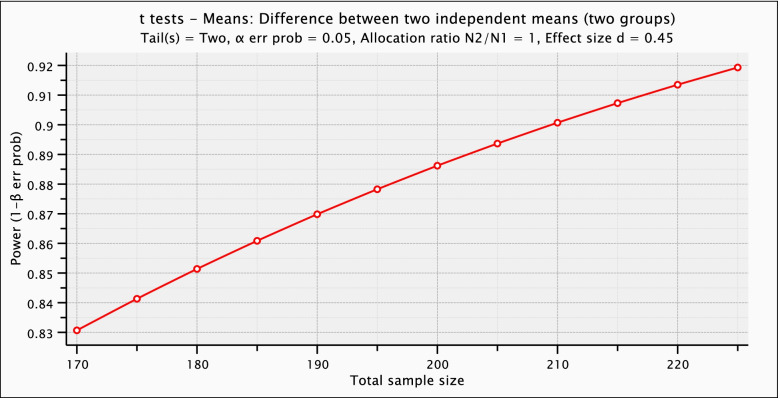
Power analysis of means difference. Analyses and graph computed using the statistical program G*Power, version 3.1.3 [20]

Following those criteria, our power analysis (Fig. 2) uses a smaller effect size of *d* = 0.45 and a total sample size between 175 patients for 84% power to 210 patients for 90% power (allocation ratio = 1, equal numbers in each group). Sensitivity analysis for a sample size of 200 patients resulted in 80% power for an effect size of *d* = 0.40, 90% power for an effect size of *d* = 0.46, and 95% power for an effect size of *d* = 0.60.

We also looked at power for the nonparametric Wilcoxon–Mann–Whitney test, in case model assumptions are not met, and found similar results.

**Fig. 3:**
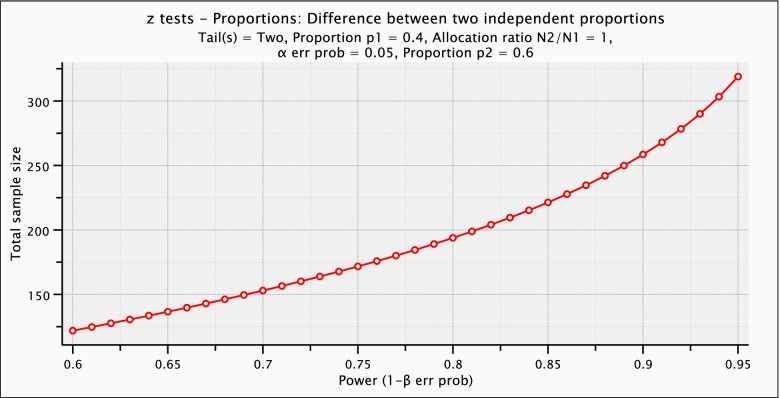
Power analysis of proportions. Analyses and graph computed using the statistical program G*Power, version 3.1.3 [20]

Aim 2 includes two secondary outcomes, Patient Quality of Engagement in Decision-Making (PQED) and Preference-Congruent Decision-Making (PCDM) (Table 1). The PQED is measured as an average difference in pre-posttest scores of four scales that are also measured as numerical scores so the above analysis will also apply to them. However, the PCDM measures in proportions the outcome of patients whose decision to screen is congruent with actually receiving or not receiving the PSA test. As this outcome is a proportion, we will have less ability to detect differences between groups. A centered difference of 20% (proportion 1 = 40%, proportion 2 = 60%) can be detected with 85% power for a total sample size of *n* = 222 and 80% power for a total sample size of *n* = 194 (Fig. 3).

For both aims, attrition will likely be negligible (< 3%) given that primary outcome will be measured during the medical encounter (aim 1) and PSA utilization data will be extracted from the electronic health record. For follow-up of PSA utilization (aim 2), the study would have 80% power to detect a between-group difference in 3-month utilization of 22% as statistically significant and 99% power to detect a utilization difference of 33% as statistically significant. These represent the most conservative estimates when accounting for the fact that power to detect a between-group difference in utilization varies based on the overall utilization rate of the entire sample. If attrition were as high as 5%, which is unlikely, the minimum effects observed as significant at 80% and 99% power would be *d* = .47 and *d* = .71 for the preference-congruent decision making (PCDM) outcome and between-group differences of 23-34% for utilization of PSA tests.

### Setting

The study is being conducted in clinics, located in the Greater New Orleans area of Louisiana, U.S., that offer annual wellness visits and preventive care to the target patient population (see the “Participant eligibility” section). Our participating clinical sites (The Tulane Medical Center and The University Medical Center of New Orleans) consist of public, private, and academically affiliated hospitals and clinics providing primary care services.

### Participant eligibility

During the study period, we expect to recruit 200 male patients who (1) self-identify as African American; (2) are 40–69 years old; (3) are currently a patient of a study clinical site; and (4) are able to read and understand spoken English (5th grade level). Patients will be excluded if they have (1) a current diagnosis or personal history of prostate cancer (ICD-10-CM codes C61 or Z85.46, respectively) or (2) medical conditions that inhibit them to complete any aspect of the intervention.

### Randomization, allocation, and blinding

Half of patient participants (*n* = 100) will be randomly assigned to the intervention arm (an educational program about prostate cancer risks, screening, and decision-making), and the other half (*n*=100) will be randomly assigned to the control arm (usual care). Randomization is conducted automatically in the data collection system (see the “Data collection and management” section) using the Qualtrics XM platform [22]. Upon signing consent and completing enrollment forms, Qualtrics generates a random 5-digit number and records it as the participant’s ID number. Participants are then able to access and complete their baseline assessments (pre-tests). Once submitted, a randomizer within the software assigns participants to the control or intervention arms, associates the assigned group to the participant ID numbers, and displays the assignment to the participant and research personnel assisting their enrollment. The PCPs conducting the medical encounters are not told if their patient is in the control or intervention arm of the study. Participant information is disassociated from their data and participant ID numbers leaving study staff assessing outcomes blinded to the randomization arms. Although there is no special criteria for discontinuing or modifying allocated interventions, patients already enrolled in the study who develop prostate cancer along the study timeline will be allowed to continue the study regarding the arm allocation. However, participating patients and clinicians who for any reason leave the clinical sites will be discontinued of their participation in the study.

### Study timeline

The total duration of the study is 5 years (April 2019–December 2023). In general, we have revised and updated the study procedures, educational intervention, recruitment strategies, and assessment materials in year 1 and began enrollment in year 2. Assessment of the comparative effectiveness of the intervention will occur during years 2, 3, and 4 (clinical trial). Follow-up of study outcomes, evaluation of the acceptability of the intervention, and preparing manuscripts for publication of results will occur in year 5.

### Decision aid (intervention)

The educational program “Prostate Cancer Screening: Making the Best Choice for You” has as the objective to enhance knowledge about the prostate gland and prostate cancer, risks, screenings, and the application of shared decision-making (SDM) skills. It was developed based on the original patient decision aid Prostate Cancer Screening: Making the Best Choice, a website interactive and accompanying pamphlet developed by Georgetown University Medical Center [23]. The training was updated, with permission of the original authors, to comprise of five videos modules able to be played within our data collection system. Each video module focuses on distinct learning objectives and includes clickable definitions, embedded animations, and fully narrated audios of all written information. At the time of automatic assignment to the intervention arm in Qualtrics, participants are given access to a survey that presents one video module per page. Participants advance to each subsequent module of the decision aid by advancing through the survey itself.

## Methods

As this study is a RCT conducting research in human subjects, the study procedures, including informed consent and protections stated by the Health Insurance Portability and Accountability Act (HIPAA), have been approved by the Institutional Review Boards (IRBs) of each clinical site before starting study activities (see the “Ethics approval and consent to participate” section). All members of the research team (principal investigators, medical residents, research assistants, and project coordinator) have completed the required training to conduct research with human participants and are authorized, by the respective IRB of each clinical site, to carry out study activities.

### Pre-screening of potential participants and invitation to join the study

Members of the research team who have credentials to access medical records, working closely with clinic staff, review patient rosters and medical records to identify all potentially eligible patients (African American men, 40–69 years old, with no prostate cancer diagnosis). Contact information (name, address, phone numbers, and emails) is extracted from the medical records into a digital file. This data is used to fill out an invitation letter and/or email that the research team sends to those eligible patients. Eligible patients who are at the clinic for other reasons are invited personally by clinical staff, and/or the research team, and given a copy of the letter, if they are interested in the study. Flyers are posted in the different clinical sites participating in the study and available to pick up in the clinic waiting rooms.

Additionally, PCPs (MDs, residents, nurse practitioners, etc.) providing primary care services to potential patients are identified through the patient medical records and their contact information (name, email, and phone) are extracted (entered in a digital file). An email introducing the study procedures and including a link to the PCP baseline survey is sent by study investigators, clinical sites directors, and/or department chairs to all the primary care providers (PCPs) identified.

### Patient enrollment, consent, and baseline (visit #1)

These steps are conducted by those members in the research team who have access to contact information of the prospective patients to be invited to join the study. The invitation letters and/or emails include basic information about the study; a personalized link to the electronic consent and baseline surveys; and contact information in case that participants want to review the consent, have questions, and/or prefer to setup a meeting (online or face-to-face) to complete the procedures with the support from the research team. Follow-up phone calls are made to those who have not enrolled.

In this visit, patients review and sign the electronic consent to participate in the study, including authorization to audio-record a clinical encounter, and HIPAA authorization to access their medical records. The electronic consent is presented on a set of questions and short answers extracted from the entire consent form and a link to the entire consent is available so participants can read and save the entire document before continuing with the consent process. Patients sign the documents by writing their name using a finger, mouse, or stylus pen.

For those patients who prefer to meet with the research team to complete the procedures, the meetings are scheduled based on participant preferences and constraints (time, transportation, literacy level, and access to computer/Internet). Online meetings are setup on Zoom, Teams, Doximity, or Skype, and face-to-face meetings are completed at the private offices of research personnel, at the respective clinic, or in a specific place chosen by the patient where privacy may be preserved. Signed copies (digital or paper) of the informed consent and HIPAA authorization forms are given to all participants to keep.

Once patients are enrolled, they complete the online baseline survey and intervention, if assigned (see the “Educational intervention procedures” section). After completing the procedures, patients receive a $40 electronic or physical gift card of their choice as compensation for the time spent and any expenses incurred during the visit.

### Educational intervention procedures (online training)

Patients randomly assigned to the intervention arm complete the educational program “Prostate Cancer Screening: Making the Best Choice for You” (see the “Decision aid” section). Once patients complete the enrollment and baseline (visit #1), the research team sends an email to those participants assigned to the intervention arm. The email includes a personalized link to the training and respective surveys, and contact information in case that participants have questions, need help to navigate the site, or prefer to setup a meeting (online or face-to-face) to complete the training.

Once study recruitment is closed, those patients randomized to the control condition (usual care) will be invited to complete the intervention (training), so they will not be at a disadvantage and will be able to receive the full benefits of participating in the study.

### Patient and primary care provider (PCP) medical encounter (visit #2)

In order to measure the application of the SDM process during the clinical setting, patients discuss the PSA screening with their respective primary care provider (PCP) during a medical wellness visit (annual checkup). Each week, research team sends a reminder (email and/or phone call) to all enrolled patients having scheduled wellness visits in that week and their respective primary care providers (PCPs). In their first reminder email, and before seeing an enrolled patient, the PCPs receive information summarizing the study protocol and a brochure detailing prostate cancer screening guidelines and the shared decision-making process. The wellness visits take place in the PCPs’ consultation rooms at the respective clinical site and are audio-recorded after patient/PCP’s consent.

At the beginning of the meeting, the patient and provider confirm that they authorize to audio-record the encounter and the research team member starts the recording and leaves the room. Once the encounter is completed the research assistant picks up the recorder and asks the provider and patient to immediately rate the application of the SDM process during the encounter. Once the encounter and evaluations are completed the PCP and patients receive the electronic or physical gift card ($50 and $40, respectively) for the time spent and any expenses incurred during the visit (transportation, parking, Internet, and phone services, etc.).

### Follow-up procedures (visit #3)

At 3 months (+ 15 days) after the medical encounter (visit #2) patients complete brief post-intervention surveys and an additional questionnaire about the acceptability of the intervention and procedures. The research team sends an email to participants with a personalized link to the follow-up surveys and including contact information in case that participants have questions. According to patient preferences and needs, these surveys may be administrated by the research team by phone or meetings (online or face-to-face). After completing the surveys, patients receive a $20 electronic or physical gift card as compensation for the time spent and any expenses incurred during the visit (transportation, parking, refreshments, Internet, etc.). Additionally, research team members check the medical records of participating patients each year to see if they have had or have not received a new PSA exam during the study period.

### Patient’s time commitment

The entire study procedures for each patient is completed in around six months, including follow-up. In general, consent signing and baseline survey (visit #1), take about 15 to 30 min to fill out. Intervention patients take around 30–45 min to complete the educational program (online training). The PCP/patient medical encounter (visit #2), takes around 1 h according to the clinical site schedules and includes the time needed for the patient to complete the respective surveys. Patients’ follow-up assessment (visit #3) take around 15–20 min.

## Outcome assessment

### Patient measures and outcomes

Patient outcomes are assessed using different measures and across different study intervals. The SPIRIT schedule of assessment and interventions (Table 2) includes three points of contact: visit #1 includes measures completed at enrollment and the randomization to the intervention or control arms. Visit #2 includes measures completed immediately after the medical encounter, and visit 3 includes measures completed 3 months post medical encounter. Patients randomized to the intervention (decisional aid) complete the training and respective assessment before the clinical encounter.

**Table 2:** Quantitative patient measures

Quantitative patient measures^**a**^	Enrollment (visit 1)	Intervention	Medical encounter (visit 2)	Follow-up (visit 3)
Timepoint	*(− 7 to − 60 days before visit 2)*	*(After enrollment and before visit 2)*	*(appt. day)*	*(90–105 days after visit 2)*
Approximate time to complete	15 to 30 min	30 to 45 min	60 min	15 to 20 min
**Enrollment:**				
Eligibility screening	X			
Invitation to join study	X			
Informed consent	X			
HIPAA authorization	X			
Baseline	X			
Allocation (randomization)	X			
**Intervention**				
Decision-aid (training)		X		
**Assessments**				
**Baseline**				
Demographics	X			
Health literacy	X			
**Primary outcomes**				
OPTION Scale [10]			X	
Shared Decision Making-patient version [11]			X	
**Secondary outcomes**				
Prostate Cancer Knowledge [13]	X	X		X
Decisional Confidence [14]	X			X
Satisfaction with Decision [15]	X			X
Decisional Self-efficacy [16]	X		X	
Intention-to-screen [17]	X	X	X	X
PSA screening test orders^b^	X		X	X
Actual receipt of PSA test^b^	X		X	X
**Tertiary outcomes**				
Acceptability and application of intervention (decision-aid)		X		
Acceptability of study procedures, patient version [18]				X

^a^Measures used in this study have been adapted from the ones found in the literature

^b^Data extracted from the electronic medical record

### Provider measures and outcomes

For providers, the SPIRIT schedule of assessment and interventions (Table 3) includes three points of contact: in visit #1, providers receive summary information about the study; give implicit consent to participate; provide demographic information; and report on their practices regarding PSA-based screening for prostate cancer. Providers joining the study receive an educational brochure with information about prostate cancer risk assessments, PSA screening guidelines, health disparities in prostate cancer and recommendation for African American men, and a summary of the SDM process and tips to apply SDM during the medical encounter. Visit #2 includes measures completed immediately after each medical encounter, and visit 3 includes the study evaluation survey completed if the provider leaves the clinical site or at study closeout. Before each medical appointment (visit #2) with a participant patient, providers receive an email with the confirmation of the appointment and a reminder of study procedures.

**Table 3:** Quantitative provider measures

Quantitative provider measures^**a**^	Enrollment (visit 1)	Medical encounter confirmation	Medical encounter (visit 2)	Follow-up (visit 3)
Timepoint	*(− 7 to − 60 days before visit 2)*	(*−* 3 to *−* 1 days before visit 2*)*	*(appt. day)*	*PCP exit or study closeout*
Approximate time to complete	*10–15 min*	5*–*10 min	*60 min*	*5–10 min*
**Enrollment**				
Eligibility screening	X			
Invitation to join study	X			
Implicit consent	X			
Baseline	X			
Educational brochure	X	X		
**Assessments**				
**Baseline**				
Demographics	X			
PSA screening practices	X			
**Primary Outcomes**				
OPTION Scale [10]			X	
Shared Decision Making-provider version [12]			X	
**Secondary outcomes**				
PSA screening orders^b^			X	X
**Tertiary outcomes**				
Acceptability of study procedures, provider version				X

^a^Measures used in this study have been adapted from the ones found in the literature

^b^Data extracted from the electronic medical record

### Operationalization of variables

#### Primary outcome

The assessment of the primary outcome, *Level of Engagement in SDM process regarding PSA-based screening*, is conducted through the rating of the audio-recorded medical encounters using the OPTION scale [10]. Two raters independently rate each conversation according to the level of competence observed in 12 communication behaviors stated in the OPTION scale. Sub-scores range from 0, representing “the behavior is not observed,” to 4, indicating “the behavior is exhibited to a very high standard.” The 12 sub-scores are added to produce an overall score from 0 to 48. The average of rating scores of the two raters are computed to get the final score. A third, independent rater, in case of need, may be used to average with the previous two scores to improve wide discrepancies in individual ratings. Additional to this independent assessment of the PCP-patient conversation using the OPTION scale, patients and PCPs report their own perceptions of the encounter using the SDM questionnaires [11, 12]. The mean scores of the patient and provider own ratings are compared to the OPTION scores and analyzed to identify differences in individual perceptions of the application of SDM process during the medical encounter versus actual performance (Table 4).

**Table 4:** Operationalization of primary outcome variables

Measures^**a**^	Operationalization of outcomes
Independent rating of SDM [10]	**12 questions (Likert scale)** • Rating: 0 to 4 • Total scale ranges from 0 to 48 • Higher values mean higher patient involvement during the SDM process
Patient rating of SDM [11]	**11 questions (Likert scale)** • Rating: 0 to 4 • Total scale ranges from 0 to 44 • Higher values mean higher involvement during the SDM process
Provider rating of SDM [12]	**11 questions (Likert scale)** • Rating: 0 to 4 • Total scale ranges from 0 to 44 • Higher values mean higher involvement during the SDM process

^a^Measures used in this study have been adapted from the ones found in the literature

#### Secondary outcomes

The study has two secondary outcomes: Patient quality of engagement in decision making (PQED) and preference-congruent decision making (PCDM).

PQED is assessed through an average difference in pre-posttest scores of four scales: Prostate Cancer and Screening Knowledge, Decisional Confidence, Decisional Self-efficacy, and Satisfaction with Decision (Table 5). Higher percentage scores mean higher quality of SDM process. Questionnaires are completed by patients in two different points of care: at enrollment (baseline) and at 3-month follow-up.

**Table 5:** Operationalization of secondary outcome variables

Measures^**a**^	Operationalization of variables
**Patient Quality of Engagement in Decision Making (PQED)**	
Prostate Cancer and Screening Knowledge [13]	**20 questions (true/false)** • Rating: 1 point for each correct answer • Total score ranges from 0 to 20 • Higher values mean higher knowledge of prostate cancer and screening
Decisional Confidence [14]	**10 questions (yes/no)** • Rating: 4 points for each “YES,” 0 points for each “NO,” 2 points for each “NOT SURE” • Total score ranges from 0 to 40 • Higher values mean higher confidence in the decision made
Decisional Self-efficacy questionnaire [15]	**4 questions (Likert scale)** • Rating: 1 to 5 • Total score ranges from 4 to 20 • Original scale scores were reversed. • Higher values mean higher patient decisional efficacy in the communication with the PCP
Satisfaction with Decision [16]	**6 questions (Likert scale)** • Rating: 1 to 5 • Total score ranges from 6 to 30 • Higher values mean higher satisfaction with the decision made
**Preference-Congruent Decision Making (PCDM)**	
Intention-to-screen [17]	**1 question (yes/no)** • Rating: 0 (no intention to screen) or 1 (intention to screen)
Actual PSA-screening tests	**1 question (yes/no)** • Rating: 1 (patient has had at least one PSA-test) or 0 (patient has NOT had PSA-tests) from medical encounter (visit #2) to follow-up (visit #3)
Congruence	**1 question (positive/negative)** Ratings: • **PCDM = 1 (POSITIVE:** congruence between patient intention and real action**)** for: • Patients with intention-to-screen score = 1 (intention = yes) and PSA-screening score = 1 (PSA = yes) • Patients with intention-to-screen score = 0 (intention = no) and PSA-screening score = 0 (PSA = no) • **PCDM = 0 (NEGATIVE:** in-congruence between intention and real action**)** for: • Patients with intention-to-screen score = 1 (intention = yes) and PSA-screening score=0 (PSA = no) • Patients with intention-to-screen score= 0 (intention = no) and PSA-screening score = 1 (PSA = yes)

^a^Measures used in this study have been adapted from the ones found in the literature

PCDM is measured as a comparison between the patient’s intention-to-screen versus the actual PSA-screenings reported in the medical records. Measures are assessed from the medical encounter (visit #2) to follow-up (visit #3). The outcome is accessed through 3 indicators: Intention-to-screen, Actual PSA-tests received, and Congruence; this is the match between intention-to-screen and actual PSA-screening behaviors (Table 5).

#### Exploratory outcomes

Exploratory outcomes include the comparative measures of acceptability of the study procedures (enrollment process, intervention, and medical encounters) and overall satisfaction with the study (Table 6). The measures are completed by patients during the follow-up (Visit #3) and by PCPs during study closeout

**Table 6:** Operationalization of tertiary outcome variables

Measures^**a**^	Operationalization of variables
Acceptability and application of intervention (decision-aid)	**3 satisfaction questions (Likert scale)** • Rating: 1 to 5 • Total score ranges from 3 to 15 • Higher values mean higher satisfaction with the training modules **5 usefulness questions (Likert scale)** • Rating: 1 to 4 • Total score ranges from 5 to 20 • Higher values mean higher acceptability with the training modules
Acceptability of study procedures, patient version [18]	**10 Likert scale questions** • Rating: 1 to 5 • Total score ranges from 10 to 50 • Higher values mean higher acceptability and satisfaction with the study procedures
Acceptability of study procedures, provider version	**5 Likert scale questions** • Rating: 1 to 5 • Total score ranges from 5 to 25 • Higher values mean higher acceptability and satisfaction with the study procedures

^a^Measures used in this study have been adapted from the ones found in the literature

### Data collection and management

To evaluate the efficacy of the decision aid, we collect quantitative data from the surveys administered to patients and providers as well as from patients’ electronic health record (Epic, eClinical, etc.). The study protocol is integrated, applicable to each independent site and approved by the respective collaborators. Step-by-step description of procedures (who does what and how) including documents and resources needed to complete each one of the study activities are described in the Manual of Procedures (MOP). All research team members receive the respective training regarding the procedures to be sure that the protocol is administrated as designed. Following the MOP, the Data Collection System (DCS) was designed to integrate the electronic collection of structured information from patients and PCPs joining the study (patient consent/HIPAA documentation, demographic data, and baseline assessments); the automatic randomization process; the respective surveys collecting data needed to measure the study outcomes; and the electronic delivery of the educational interventions (prostate cancer screening decision aid); as well as the electronic distribution of incentives (gift cards). The DCS includes quantitative and qualitative information that is collected not only when PCPs and patients complete the study activities but also during the support given by research personnel during the recruitment process, medical encounters, and follow-up surveys. The DCS was developed using Qualtrics XM platform, versions 2020-2022. Qualtrics is a powerful cloud-based customer experience management program that allows creating and distributing surveys online. It has the capability to automatically code, validate, and process data, as well as automatically run basic statistics and produce reports [22].

The grantee, Xavier University of Louisiana, is responsible for developing and maintaining the study password-protected online data collection system, including the measures stated in the IRB protocol, and coding schemas. As owner of the data, Xavier is responsible for the security of data access and privacy, and the implementation of the controls stated in the respective IRB protocol. Any changes in the measures require the approval by the study lead principal investigator (PI) and are appropriately documented in the “summary of changes and version control” table that is included at the beginning of each IRB document.

### Study monitoring and safety plan

This is an educational intervention where participants read materials, watch videos and complete questionnaires and pre-post assessments. Considering that participation in this research presents no more than minimal risk and no life-threatening events, there are no adverse events anticipated in this study. The research team meets weekly to review/discuss any issue related to the study, including the inclusion and enrollment report. As members of the research team, the project coordinator and the medical residents and research assistants in each clinical site are responsible for the day-to-day support for the trial procedures including participant screening, recruitment, consent, and retention along the study timeline. The principal investigator and co-investigators in each clinical site are responsible for supervising the trial including data safety and monitoring and report, directly to the respective IRBs, any participant’s issue that may be related to participation in the research and that may pose a greater risk than previously recognized. The External Advisory Board (EAB) follow-ups the study and conducts two official reviews per year. The IRB revises the study reports and advance once per year. Both the EAB and IRB have authority to extend study activities and/or stop, suspend, or require modifications to the study, if needed. The study statistician is responsible for the production of interim analysis, including stopping points, and recommendations to terminate the trial, if needed. In addition, the Patient Advisory Board (PAB) provides revisions and feedback regarding study interventions, recruitment materials, and survey procedures. The PAB is formed by African American men, some of them being prostate cancer survivors, who represent the communities targeted in the study. The PAB members meet twice per year to review the advance of the study, follow-up with participants, and give recommendations on how to address specific patient and community needs regarding early detection of prostate cancer.

Qualtrics, the data collection system used in this study to capture and store data, is HIPAA-compliant, uses Transport Layer Security (TLS) encryption for all transmitted Internet data, has the functionality to ensure the confidentiality of survey results, and has data auditing to ensure any changes to data are recorded [22]. Access to study records is limited to IRB-approved members of the study team. The specialist in Qualtrics support is responsible for data protection, creation of user accounts including privileges to access study raw data, and maintenance of the Qualtrics system at the university level.

### Statistical analysis

Preliminary analysis, prior to hypothesis testing, focuses on descriptive statistics of measures of frequency (count, percentage, frequency), central tendency (mean, median, mode), and dispersion (range, variance, standard deviation) to summarize the characteristics of the sample, check assumptions underlying analytic procedures, and evaluate whether randomization produced comparable patient groups on demographics (age, insurance, family PrCa history, previous PSA utilization, education, and health literacy level) and baseline scores. Exploratory/confirmatory factor analyses will be used to examine and report on the psychometric properties of all study measures.

Hypothesis testing for the Level of Engagement in SDM (primary outcome) and for Patient Quality of Engagement in Decision-Making (PQED) include two-tailed hypothesis tests with an α-level of 0.05. ANOVAs and chi-square tests will be used to assess group differences on scale scores using controlling procedures to account for multiple comparisons (Holm, Bonferroni, or other procedures). Although the intervention is tailored based on education and prostate cancer family history, it is possible these variables may nonetheless account for some variability in learning from the intervention. Family history of prostate cancer may be associated with increased familiarity with or a desire to have PSA screening. Controlling for the baseline status on outcome variables, where possible, reduces within-patient variance in effect estimates. We will conduct sensitivity analyses for the PQED subscales separately to identify predictors of missing data and characterize the robustness of the effects observed.

Analysis for preference-congruent decision making (PCDM) consist of binary logistic regressions to examine the impact of the intention-to-screen (dichotomous independent variable) on PSA utilization (dichotomous outcome) while controlling for covariates.

We will run separate analysis of intervention and control arms to examine possible associations between separate measures (Prostate Cancer and Screening Knowledge, Decisional Confidence, Decisional Self-efficacy, and Satisfaction with Decision) and the study primary (SDM) and secondary (PQED, and PCDM) outcomes.

In general patients’ age range, health insurance type, family prostate cancer history, previous PSA utilization, education level, and intention-to-screen at baseline (visit 1) as well as their PCPs’ gender, age, race, clinical site, and specialty (internal medicine, family medicine, etc.) will be explanatory variables under consideration in all analysis.

## Discussion

The proposed research is timely and highly innovative in that it responds to the U.S. Preventive Services Task Force (USPSTF) May 2018 final recommendation that male patients and their providers engage in a shared decision-making process about the benefits and harms of PSA-based screening for early detection of prostate cancer. These conversations are especially important for African American men, given the increased ambiguity due to the lack of PSA-based research specific to this population and increased risk of prostate cancer mortality attributed to late stage at diagnosis and more aggressive prostate cancer phenotypes seen in African American men. In our study, the shared decision-making intervention has the potential to reduce racial health disparities on several fronts. One, we will follow up on the results of PSA-based screening among African American men who, based on informed preferences, want to be screened. For some men with highly elevated PSAs, the intervention may lead to earlier diagnosis and treatment, mitigating race-based disparities in late-stage diagnosis and potentially reducing racial disparities in prostate cancer mortality. Two, by making PSA-based screening a shared decision based on patient preferences and risk, rather than one driven by practice-level default norms or implicit biases, services will be focused on reducing the burdens of unnecessary care, while reducing disparities in morbidity for those with higher risks. Three, if effective compared to usual care, the intervention will have significant impacts in increasing quality of healthcare services provided (higher satisfaction and quality of decisions implies higher patient satisfaction with services) and increasing access to and utilization of services that patients prefer, thereby improving the overall quality of life aspect of health disparities.

## Trial status

The trial was registered prospectively with the National Institute of Health registry (https://clinicaltrials.gov/ct2/show/NCT03869216), registration number NCT03869216, on March 11, 2019. This trial is ongoing. Recruitment began on August 17, 2020, and will continue until September 2023. The trial procedures are expected to be completed by the end of March 2024.

Protocol version 02.26.20.

## Supplementary Information

**Additional file 1.** SPIRIT Checklist

## Data Availability

This study will comply with the NIH Public Access Policy, which ensures that the public has access to the published results of NIH funded research. As required, we will submit final peer-reviewed journal manuscripts to the digital archive PubMed Central upon acceptance for publication. De-identified summary of participant data for all primary and secondary outcomes measures, and supporting trial materials will be made available within 6 months of study completion. Data access request will be reviewed by the full IRBs and principal investigators. If approved, requestors will be required to sign a Data Access Agreement.

## Abbreviations

AAM: African American men
HIPAA: Health Insurance Portability and Accountability Act
IRB: Institutional Review Board
*N*: Number (typically refers to number of participants)
NIH: National Institutes of Health
PI: Principal Investigator
PCP: Primary care provider
PCDM: Preference-congruent decision making
PQED: Patient Quality of Engagement in Decision-Making
PrCa: Prostate cancer
PSA: Prostate-specific antigen
RCT: Randomized clinical trial
RCMI: Research Centers of Minority Institutions
SDM: Shared decision-making
U.S.: United States
